# Integrated metabolomic and transcriptomic analysis reveals novel plasma biomarkers and metabolic pathway dysregulation in latent tuberculosis infection

**DOI:** 10.1128/spectrum.03482-25

**Published:** 2026-03-27

**Authors:** Dong Liu, Zhelong Feng, Xiangrui Meng, Yanhan Li, Yuerong Zeng, Yan Li, Fang Yan, Yajing Wang

**Affiliations:** 1 Department of Clinical Pharmacy, School of Basic Medicine and Clinical Pharmacy, China Pharmaceutical University 540422, Nanjing, China; 2 Integrated Service & Management Office, Jiangsu Provincial Center for Disease Control and Prevention 12666, Nanjing, China; 3 Department of Pharmaceutical Analysis, School of Pharmacy, China Pharmaceutical University 630740, Nanjing, China; Tainan Hospital Ministry of Health and Welfare, Tainan, Taiwan, Province of China

**Keywords:** latent tuberculosis infection, metabolomics, multi-omics, biomarkers

## Abstract

Tuberculosis (TB) remains a global health threat, and latent TB infection (LTBI) serves as a significant reservoir for new cases. Current diagnostic methods have limitations in accuracy and specificity. This study aimed to identify novel plasma biomarkers for LTBI by integrating metabolomics and transcriptomics. Plasma samples from healthy controls (HC) and LTBI patients were analyzed using liquid chromatography-mass spectrometry (LC-MS)/MS. We identified distinct metabolic signatures characterizing LTBI, and a diagnostic model based on five key metabolites—margaroylglycine, N-palmitoyl tryptophan, oleamide, myristic acid, and pentadecanoic acid—was established, demonstrating discriminatory potential. Multi-omics integration revealed that these metabolites are significantly associated with perturbations in glycerophospholipid and tryptophan metabolism pathways. Validation via qPCR confirmed significant dysregulation of associated genes (*PLA2G4C*, *PLA1A*, *CDS2*, *IL4I1*, *EHHADH*, *IDO2*, and *ASMT*) in peripheral blood mononuclear cells (PBMCs). External validation in independent cohorts confirmed the consistency of these metabolic alterations. These findings suggest a robust panel of biomarkers for LTBI screening and offer exploratory insights into the metabolic dysregulation underlying latent infection.

**IMPORTANCE:**

Latent tuberculosis (TB) infection (LTBI) acts as a silent reservoir for the global tuberculosis epidemic, yet current diagnostic tools often lack the specificity to fully capture the host's biological response. This study addresses this critical gap by using a multi-omics approach to decode the hidden metabolic changes in the blood. We discovered a unique “chemical fingerprint” consisting of five specific metabolites that can accurately distinguish latent infection from healthy states. Beyond providing a highly accurate diagnostic model, our findings reveal how the infection subtly reprograms host lipid and immune pathways. This work is significant because it offers a promising new tool for clinical screening and deepens our understanding of the metabolic interplay between the host and the pathogen, paving the way for better disease control strategies.

## INTRODUCTION

Tuberculosis (TB), caused by *Mycobacterium tuberculosis* (M.tb), is a respiratory infection that significantly threatens human health, primarily affecting the lungs (1). In 2021, China reported 780,000 new TB cases, ranking third among the 30 high-burden TB countries, following Indonesia and India. According to the WHO’s Global Tuberculosis Report 2022, individuals with latent tuberculosis infection (LTBI) contribute substantially to the emergence of new TB cases. LTBI occurs when the immune system contains M.tb infection without manifesting active disease symptoms (2). Current diagnostic methods, including the Tuberculin Skin Test (TST) and Interferon-Gamma Release Assays (IGRAs), have limitations such as false positives and an inability to distinguish latent from active pulmonary TB (ATB) or predict disease progression. Therefore, accurate and timely detection of LTBI is critical for controlling the TB epidemic.

TB not only induces pathological changes in the lungs but also causes systemic metabolic disturbances, affecting energy metabolism and other physiological processes. Metabolites, which reflect the cellular environment more accurately, often alter before clinical symptoms appear, making metabolomics a valuable tool for understanding disease mechanisms and improving clinical diagnoses. Furthermore, metabolomic studies have the potential to uncover biomarkers for TB diagnosis (3, 4). However, research on the metabolomics of TB, particularly LTBI, remains limited, with most studies focusing on ATB. Yufeng Yao’s team identified 17 significantly altered metabolites in patients with TB, including 1-methylhistidine, pyruvate, isoleucine, and acetoacetate. These changes suggest increased energy expenditure, enhanced glycolysis, disrupted fat metabolism, and altered amino acid and nucleotide metabolism in patients with TB (4, 5). Subsequent analyses of plasma from patients with diabetes, malignancies, and pneumonia confirmed the specificity of these metabolites to TB caused by M.tb infection (6). Che et al. utilized GC/TOF-MS to analyze serum from patients with TB and identified 5-oxoproline as a potential early diagnostic marker, although its mechanism remains unclear (7). I du Preez et al. observed that M.tb infection significantly upregulated the glyoxylate cycle, accelerating the citric acid cycle and enhancing fatty acid and cholesterol consumption (8). Shuang Feng et al. conducted a simultaneous analysis of plasma from patients with active pulmonary TB, patients with lung cancer, and healthy controls (HC), constructing a TB-specific metabolic profile and identifying 12 differential metabolites, including fatty acids, amino acids, and lipids (9). Susanna et al. employed LC-MS to identify 4 novel plasma biomarkers in patients with TB compared to healthy individuals, including ceramide (d18:1/16:0), cholesterol sulfate, and arachidonic acid (10). Despite these advances, metabolomics research on LTBI remains sparse, and effective biomarkers for controlling TB transmission are still lacking.

Multi-omics approaches have advanced the discovery of novel biomarkers for TB. Petros C Karakousis et al. employed transcriptomics and metabolomics to examine differentially expressed miRNAs and metabolites in patients with TB treated with anti-TB drugs versus HIV-TB co-infected patients on isoniazid prophylaxis. Their analysis identified hsa-miR-215-5p and γ-glutamylsulfonic acid as markers targeting the TGF-β signaling pathway, effectively distinguishing the two patient groups (11). Ana Varela Coelho et al. applied metabolomics to identify five biomarkers—9-methyluric acid, trans-3-indoleacrylic acid, indole-3-lactic acid, hexanoylglycine, and N-acetyl-L-leucine—along with proteomics, which highlighted hemopexin and revealed the involvement of the complement system and cholesterol, iron, and tryptophan metabolism in TB pathophysiology (12). Wang et al. combined proteomics and miRNA transcriptome sequencing to analyze serum from patients with multidrug-resistant TB and healthy controls, identifying CD44, KNG1, miR-4433b-5p, miR-424-5p, and miR-199b-5p as key biomarkers for a diagnostic model (13). However, these biomarkers require further validation due to potential overlap in biological alterations seen in other diseases. Moreover, there is a pressing need for deeper exploration into metabolic pathways and biomarkers specific to LTBI.

This study aimed to investigate metabolic changes during LTBI, identify differential metabolites associated with LTBI, and elucidate the mechanisms of metabolic dysregulation. A logistic stepwise regression model was developed to select potential biomarkers from the identified metabolites. Integrated transcriptomic and metabolomic analyses were subsequently conducted to pinpoint metabolic pathways linked to correlated differential genes and metabolites. Finally, qPCR was employed to validate the expression of target genes within these pathways. The findings provide a basis for novel molecular biomarkers for LTBI diagnosis and enhance the understanding of the metabolic changes and pathological mechanisms underlying LTBI.

## MATERIALS AND METHODS

### Sample collection

Blood samples were collected between March and November 2021 in Xuzhou, Yangzhou, Huai'an, and Yancheng, Jiangsu Province. All participants were close contacts of patients with ATB, and blood samples were obtained after fasting for QuantiFERON-TB Gold (QFT) testing. Based on the QFT results, 81 patients with LTBI (QFT-positive) and 85 healthy controls (QFT-negative) were selected. At the time of peripheral blood collection, clinical information such as name, age, gender, relevant clinical biochemistry, pathological test results, and clinical diagnosis was recorded under the participant’s ID number.

### Sample preparation

Peripheral venous blood was collected using heparin lithium anticoagulant vacuum tubes. Three milliliters of blood was used for QFT testing, while the remaining whole blood was gently mixed and centrifuged (3,000 rpm, 10 min). After centrifugation, plasma and blood cell layers were separated. The supernatant was collected and transferred to cryovials, which were sealed and stored at −80°C for future use.

### Metabolite extraction

To eliminate large molecules, such as proteins, from plasma, a standard protein precipitation method commonly used in metabolomics was applied. Metabolites were extracted using a 1:1 mixture of methanol and acetonitrile and analyzed by liquid chromatography-mass spectrometry (LC-MS). Plasma samples stored at −80°C were thawed on ice (limited to a single freeze-thaw cycle to minimize degradation), and 100 μL of each sample was transferred to a new 1.5 mL centrifuge tube. To this, 300 μL of pre-chilled (4°C) methanol/acetonitrile mixture was added. The mixture was vortexed for 30 s and sonicated in an ice-water bath for 10 min. The samples were incubated at 4°C overnight to ensure complete protein precipitation while maintaining low-temperature conditions to preserve metabolite stability. Afterward, the samples were centrifuged at 12,000 rpm for 10 min at 4°C, and the supernatant (200 μL) was collected and freeze-dried. The freeze-dried powder was stored at −80°C. For reconstitution, 100 μL of 5% methanol in water (vol/vol) containing internal standards (L-2-chlorophenylalanine and ketoprofen, 1 μg/mL) was added to the powder. The mixture was vortexed for 3 min, centrifuged at 15,000 rpm for 10 min at 4°C, and the supernatant was collected for analysis. Additionally, 20 μL from each plasma sample was pooled into a single centrifuge tube to prepare quality control (QC) samples, which were processed similarly. A blank sample consisting of 5% methanol in water (vol/vol) was also included in the LC-MS analysis.

### High-performance liquid chromatography quadrupole time-of-flight mass spectrometry (HPLC-Q-TOF-MS)/MS

Metabolite detection was conducted using a Shimadzu LC-MS 9030 Ultra-Performance Liquid Chromatography coupled with a Quadrupole Time-of-Flight mass spectrometry (UPLC-Q-TOF-MS) system. Separation of metabolites was achieved on a Waters ACQUITY UPLC HSS T3 column (2.1 mm × 100 mm, 1.8 μm) equipped with a pre-column. The flow rate was maintained at 0.4 mL/min, with the column temperature set at 40°C and that of the autosampler at 4°C. The injection volume was 5 μL. The mobile phase A for the positive ion mode consisted of 0.1% formic acid in water, while for the negative ion mode, it was 10 mmol/L ammonium acetate in water. Mobile phase B was acetonitrile for the positive ion mode and methanol for the negative ion mode. Following UPLC separation, data acquisition was performed in the data-dependent acquisition (DDA) mode using a high-resolution mass spectrometer to collect MS and MS/MS data within a 0.5–17.5 min window. Each sampling interval included 1 full scan and 10 MS/MS scans. Background peaks were subtracted using a blank solvent, and the 10 ions with the strongest response after each full scan were selected for MS/MS analysis. The collision energy (CE) for negative ion acquisition was set to 25 ± 15 eV, and for positive ion acquisition, it was set to 30 ± 15 eV. Electrospray ionization (ESI) parameters following T3 chromatographic separation were as follows: capillary voltage of 4,000 V for the positive ion mode and −3,000 V for the negative ion mode. The flow rates for the heating, nebulizing, and drying gases (all nitrogen) were 10 L/min, 3 L/min, and 10 L/min, respectively. The temperatures for the ion source, DL tube, and heating block were 300°C, 250°C, and 400°C, respectively. To ensure system stability and data quality, a rigorous QC protocol was implemented: (a) prior to analysis, a full scan of a blank sample was performed to establish the DDA background, which was automatically subtracted. (b) Before analyzing the study samples, the LC-MS system was equilibrated by injecting 3 blank samples followed by 3 QC samples. (c) During the analytical run, 1 blank sample and 1 QC sample were inserted after every 10 study samples to evaluate instrument stability and monitor signal drift. (d) Additionally, DDA analysis of QC samples was performed at the midpoint and end of the sample queue across 4 mass ranges (*m/z* 100–200, 200–300, 300–500, and 500–1,000) to ensure comprehensive coverage.

### Untargeted metabolomics analyses

The raw mass spectrometry data were converted to .mzML format using LabSolutions software and processed using the XCMS package (version 3.2) in R (version 4.2.1). The data preprocessing pipeline included the following critical steps to correct for instrument drift and batch effects:

Noise Reduction: Noise from sample preparation or instrument interference was removed using matched filtration and moving average filtration.

Baseline Correction: Baseline shifts caused by instrument instability were corrected by subtracting the minimum value from the spectrum intensity to reset the baseline to zero.

Deconvolution: Mathematical algorithms were applied to resolve overlapping peaks co-eluting due to similar retention times or broad peak widths.

Peak Alignment (Retention Time Correction): To correct for retention time shifts caused by instrument drift or variations in sample pH/concentration, peak alignment was performed to ensure that the same metabolite features across all samples were aligned to a consistent retention time.

Peak Identification and Feature Extraction: Peak start and end points were determined, and peak features were extracted by calculating peak height or peak area. Following these correction steps, the resulting data matrix was subjected to normalization and statistical analysis.

The MetEx package (version 1.4.0) in R was then used to interpret the MS/MS data from the raw mass spectrometry output. Metabolites were annotated and identified by comparing the metabolic features to the HMDB database and the MetEx standard library, using both the MoNA and MetFrag MS/MS databases for matching the primary and secondary mass spectra. To validate the identity of the potential biomarkers, we purchased authentic chemical standards for myristic acid, pentadecanoic acid, and oleamide. Targeted multiple reaction monitoring (MRM) assays were developed to confirm their retention times and precursor-to-product ion transitions. The standard solutions were analyzed using the same chromatographic conditions as the samples. The optimal CE and ion transitions were determined for each standard (Fig. S3 to S5).

### Statistical analysis

Following metabolite annotation, the resulting three-dimensional numerical matrix underwent multivariate statistical analysis using the MetaboAnalyst 5.0 online platform (https://www.metaboanalyst.ca/). Dimensionality reduction and statistical analysis were performed using the Statistical Analysis module, where missing values in the raw data were simulated and imputed *via* the feature-dependent KNN method. Data were standardized through total sum normalization, log transformation, and Pareto scaling. After preprocessing, principal component analysis (PCA) was employed to filter out more concentrated multivariate data. To ensure the robustness and generalizability of the diagnostic models, the 166 samples were divided into a training set (*n* = 100) and a validation set (*n* = 66) using a stratified random sampling method. This strategy ensured that the distribution of key demographic variables (age and gender) remained statistically balanced between the 2 sets (*P* > 0.05), thereby minimizing potential selection bias. Supervised discriminant analysis, specifically orthogonal partial least squares discriminant analysis (OPLS-DA), was applied to highlight intergroup differences, with seven-fold cross-validation used to assess model stability and predictive power. A permutation test was conducted to check for overfitting. Variable importance in projection (VIP) scores were derived from the OPLS-DA model to identify significant intergroup differences and the relevance of each variable to the experimental groups. Variables with VIP scores greater than 1.5 were selected as significant for distinguishing between the LTBI and HC groups. A Student’s *t*-test was subsequently performed for intergroup comparisons. To address the issue of multiple hypothesis testing, the *P*-values were adjusted using the Benjamini-Hochberg (BH) method to control the false discovery rate (FDR). Features that met both an FDR-adjusted *P*-value (q-value) < 0.05 and VIP scores > 1.5 were defined as differential metabolites (Table S3). The final results were visualized in a volcano plot using R software (version 4.2.1). Differential metabolites were then imported into MetaboAnalyst 5.0 for metabolic pathway enrichment analysis through the Pathway Analysis module, aiming to identify differential metabolic pathways between the LTBI and HC groups. Based on the differential metabolites, a representative set of characteristic metabolites was selected by combining pathway analysis and biological function predictions. A receiver operating characteristic (ROC) curve was plotted using MedCalc (19.0.7) to analyze the area under the curve (AUC), sensitivity, and specificity of the selected candidate biomarkers. A binary logistic regression model was fitted to evaluate the diagnostic value of the combined model, with the AUC ranging from 0.5 to 1.0; values closer to 1.0 indicated better diagnostic performance.

### GEO data mining and analysis

The data set GSE98461, pertaining to LTBI, was retrieved from the NCBI GEO database. This data set included 12 samples: 4 from individuals with ATB, 4 from those with LTBI, and 4 from healthy controls. The experimental platform utilized was GPL6480, the Agilent-014850 Whole Human Genome Microarray 4x44K G4112F (Probe Name Version). For the comparative analysis, samples from the LTBI and healthy control groups were selected. The raw data were processed in R, with filtering criteria set at |logFC| > 1 and *P* < 0.05, followed by the generation of a volcano plot. To integrate transcriptomic differential genes with nontargeted metabolomic differential metabolites in metabolic pathway analysis, the Joint Pathway Analysis module of the MetaboAnalyst 5.0 platform was employed. Differential genes and metabolites enriched in common metabolic pathways were identified, and Spearman’s rank correlation coefficients and *P* values were computed using the psych package in R (version 4.2.1). Metabolite-mRNA pairs were filtered based on a threshold of *P* < 0.05 and |r| ≥ 0.8. A correlation heatmap was then visualized with the heatmap package in R (version 4.2.1), and a correlation network was constructed using Cytoscape (version 3.9.1). Additionally, KEGG pathway enrichment analysis was conducted for the differentially expressed genes (DEGs) within the shared metabolic pathways using the clusterProfiler package in R. Pathways with a *P*-value < 0.05 were considered significantly enriched. Finally, metabolic pathways enriched from the DEGs were intersected with those enriched from nontargeted metabolomics data to identify pathways exhibiting significant changes in both the transcriptome and metabolome. The target genes and associated metabolites in these pathways were visualized as a co-expression network using Cytoscape (version 3.9.1).

### External validation in an independent cohort

To verify the robustness of the 5-metabolite panel, we utilized two independent data sets: Lau et al. (10) (GC-MS platform) for fatty acid validation and ST002428 (LC-MS platform) for amide, acylglycine, and amino acid validation. For metabolites not directly annotated in external data sets (e.g., margaroylglycine), we targeted their chemical class representatives (e.g., hexanoylglycine for acylglycines) to validate the underlying pathway dysregulation. Diagnostic performance was assessed using AUC analysis.

### Real-time quantitative PCR

Whole blood samples were mixed and centrifuged at 3,000 rpm for 15 min. The upper plasma layer was extracted and stored in a cryotube. The plasma was then mixed with 800 μL of phosphate-buffered saline (PBS), and 10 mL of the mixture was transferred into a centrifuge tube. Subsequently, 3 mL of Ficoll was added, and the mixture was gently layered along the tube wall using a Pasteur pipette. The Ficoll layer underwent centrifugation at 1,000 *g* for 25 min, with acceleration and deceleration rates set to 1. After centrifugation, the intermediate white layer, designated as peripheral blood mononuclear cells (PBMCs), was collected. RNA extraction was performed on 8 corresponding negative and positive PBMC samples using RNA-easy Isolation Reagent (Vazyme R701-01). The extracted RNA was reverse-transcribed with HiScript II, utilizing the first-strand cDNA synthesis kit (Vazyme R211-01). Real-time quantitative PCR was conducted with AceQ Universal SYBR qPCR Master Mix (Vazyme Q511-02). The primers for the investigated genes were as follows: GAPDH-F: CGATGCCCCCATGTTTGTGA, GAPDH-R: GAGCCCTTCCACAATGCCAA; ASMT-F: AGGAGGTCTGGAGCGTCAAC, ASMT-R: GTCCCACCAAGGTCACACATAAG; IL4I1-F: AAGGCACACGCTCTTGGAATATC, IL4I1-R: AGAAGCCATCCTCGGACATCAC; EHHADH-F: TCAGTTGGTGTTGTTGGCTTGG, EHHADH-R: CGAGTCTACAGCAATCACAGGAATC; IDO2-F: TTATGTCTGGCAGGAAGGAGAGG, IDO2-R: CCAGTTCGTCAGCACCAAGTC; PLA2G4C-F: GGTTCACCTCATCCTCTCCTTCG, PLA2G4C-R: GGCGGCGGCAGTAGTCAG; PLA1A-F: AAGGATAGGACTGGTGGAACAAGG, PLA1A-R: GCACTGGAAGTAGTCAGGAGGTAG; CDS2-F: TGAGCCCTCGGACCTGTTTC, CDS2-R: GCGATGCTGTGAATCTGGAAGG.

## RESULTS

### Determination of plasma metabolites in HC and LTBI groups by untargeted metabolomics

**Fig 1:**
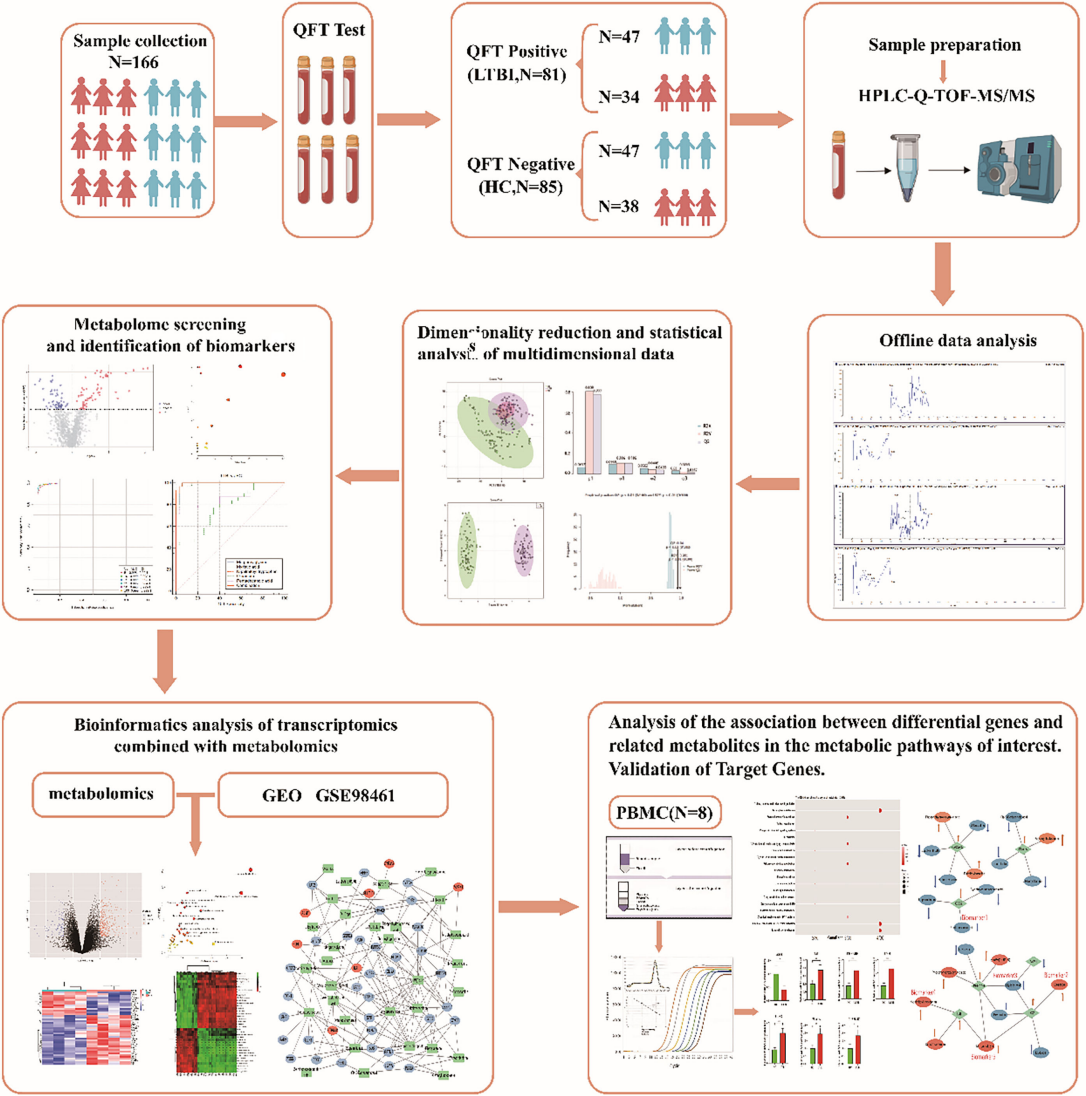
The flowchart of the entire experiment.

The flowchart of the entire experiment is shown in Fig. 1. After baseline filtering, retention time correction, peak identification, peak extraction, peak integration, peak alignment, and normalization, a total of 2,407 metabolic features were identified in the negative ion mode, and 3,070 metabolic features were identified in the positive ion mode. The data preprocessing resulted in a three-dimensional numerical matrix consisting of mass-to-charge ratio (m/z) values, retention time (RT), and peak intensity. The metabolic features from both ion modes were then combined into a single numerical matrix, which was exported to an Excel spreadsheet for further analysis (Table S1).

**Fig 2:**
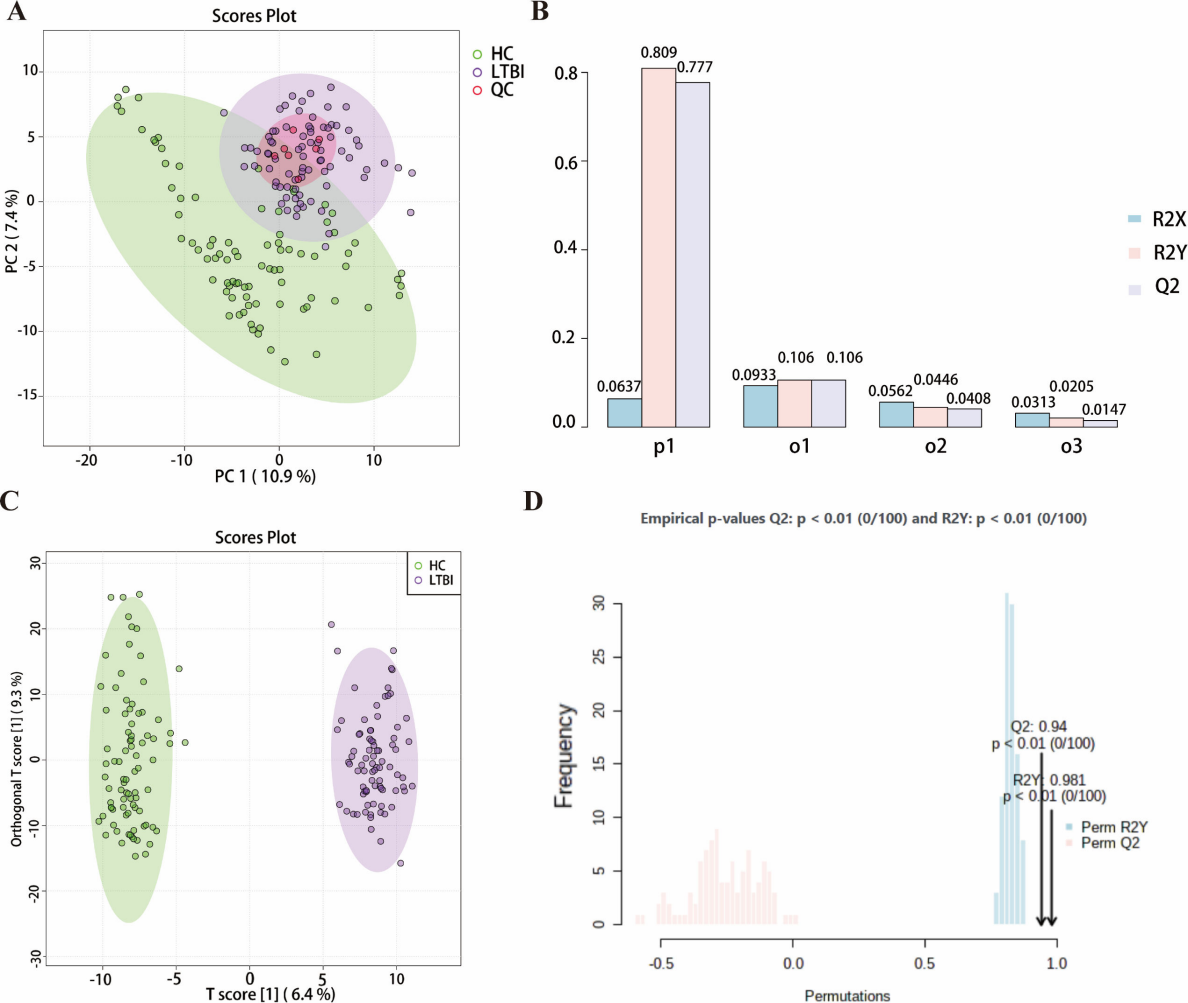
Significant differences in metabolomic profiles between HC and LTBI groups. (**A**) PCA score plot of overall metabolic characteristics (including QC samples). In the PCA plot, green represents the healthy control group, purple represents the latent tuberculosis infection group, and red represents the QC samples. (**B**) Results of seven-fold cross-validation. (**C**) OPLS-DA score plot, where green represents the HC group and purple represents the LTBI group. (**D**) Model validation permutation test results. The horizontal axis indicates the accuracy of the model, and the vertical axis represents the frequency of the accuracy of 100 models in the 100 permutation tests.

Significant differences were observed between the metabolomic profiles of the HC and LTBI groups. To ensure the reliability and quality of the metabolomic data, a QC sample was used to monitor the consistency of the analytical process. PCA suggested that concentrated QC samples reflected the reliability of the data. The results indicated that all QC samples clustered near the origin, suggesting stable instrument performance and high-quality signals (Fig. 2A). These results confirmed minimal instrument error, stable data, and reliable experimental repetition, paving the way for further statistical analysis of metabolic profile differences. While the LTBI and HC groups were not completely separable, partial distinction was observed, suggesting metabolic differences between the two groups. Therefore, supervised statistical analysis was employed for subsequent data processing.

To further highlight the metabolic small molecules with significant differences between the LTBI and HC groups, an OPLS-DA model was constructed based on the PCA results. The DA model excluded orthogonal variables unrelated to the classification variable, enabling a detailed analysis of group differences. The OPLS-DA assessment plot indicated a clear separation of the metabolic profiles of the LTBI and HC groups (Fig. 2C). A seven-fold cross-validation method was applied, dividing the data set into seven parts, with each part selected once for validation and used to train the model. This process was repeated until all samples were predicted. The calculated cross-validation parameters, R2Y = 0.809 and Q2 = 0.777, both exceeding 0.4 (Fig. 2B), indicated that the model had strong predictive and explanatory power, accurately reflecting the data and ensuring the reliability of the analytical results.

To further assess model robustness, a permutation test (randomization test) was performed as an external verification method to check for overfitting. After 100 random permutations, the R2Y and Q2 values were 0.981 and 0.94, respectively, with a *P*-value less than 0.01 (Fig. 2D). These results indicated that the model for the LTBI and HC groups’ plasma samples adhered to parameter standards, confirming the absence of overfitting and confirming its excellent reliability, making it a suitable tool for biological marker screening.

### Metabolite identification in HC and LTBI plasma samples and metabolic pathway analysis of differential metabolites

**Fig 3:**
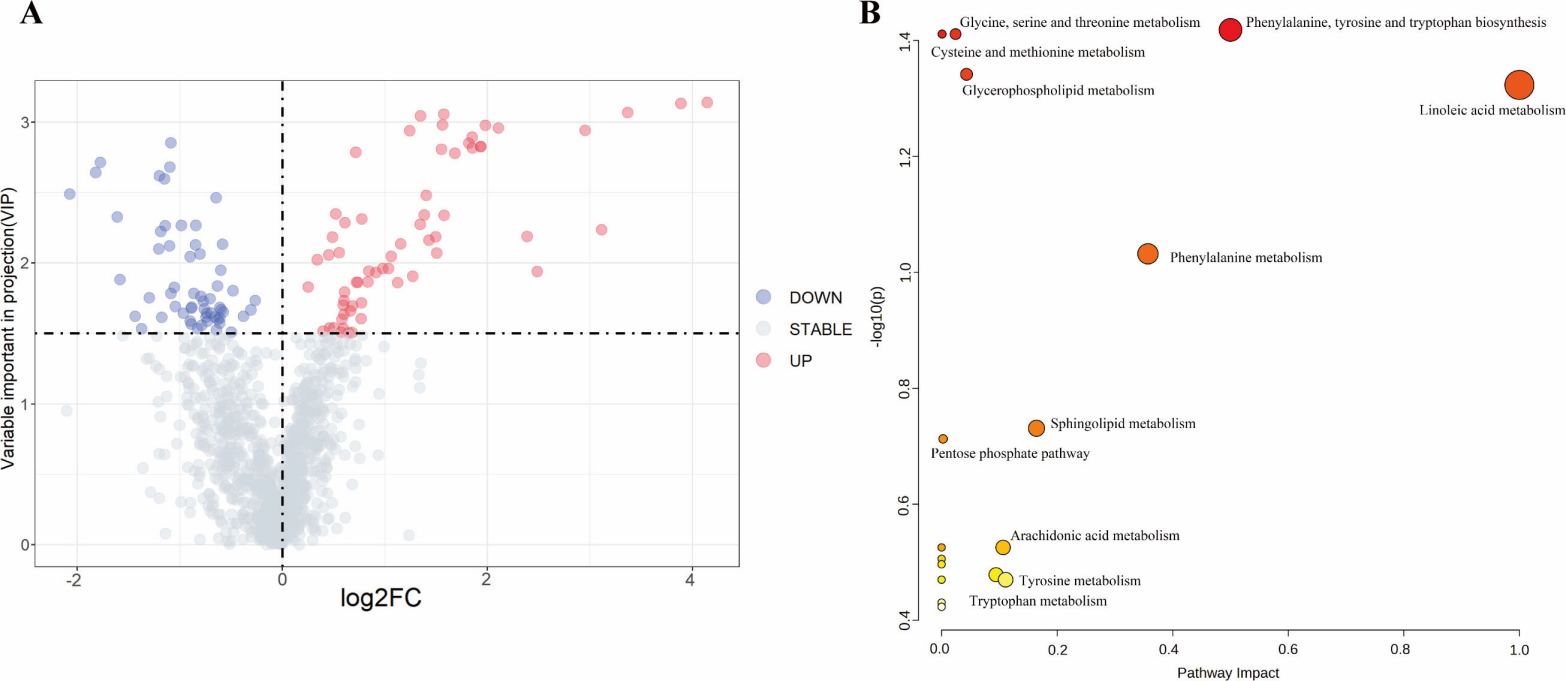
Metabolite identification in HC and LTBI plasma samples and metabolic pathway analysis of differential metabolites. (**A**) Volcano plot of differential metabolites. Red dots indicated significantly upregulated metabolites, blue dots indicated significantly downregulated metabolites, and gray dots represented metabolites without significant differences. (**B**) Bubble plot of differential metabolite pathway analysis. Metabolic pathways enriched with differential metabolites in the LTBI group and HC group. Each bubble represented a metabolic pathway. The x-axis and bubble size indicated the impact factor of the pathway in the topological analysis, while the y-axis and bubble color represented the enrichment analysis *P*-value (negatively log-transformed with base 10).

After processing the data matrix, metabolites were annotated based on their VIP values, derived from the OPLS-DA model. Metabolites with VIP values greater than 1 were considered significant for differentiating between the LTBI and HC groups. A Student’s *t*-test was performed to assess group differences, with a significance threshold set at a *P*-value of less than 0.05 and a VIP value greater than 1.5. This analysis identified 115 differential metabolites, including 52 decreased and 63 increased in the LTBI group relative to the HC group (Fig. 3A; Table S2).

The differential metabolites may exhibit overlapping or complementary biological functions and could be regulated through positive or negative feedback mechanisms within shared metabolic pathways. To explore these pathways, the MetaboAnalyst 5.0 online platform was utilized for pathway analysis, identifying key metabolic pathways associated with the differential metabolites. This approach linked metabolic substances to their corresponding regulatory proteins, providing a holistic view of cellular physiological and biochemical processes. Pathways were considered statistically significant if the *P*-value was below 0.05.

The analysis highlighted that the differential metabolites were primarily involved in pyrimidine biosynthesis, encompassing amino acids such as orotic acid, threonine, and methionine. A total of 17 metabolic pathways were identified, including methionine metabolism, cysteine metabolism, glycerolipid metabolism, arachidonic acid metabolism, beta-oxidation, phospholipid metabolism, alpha-linolenic acid metabolism, tyrosine metabolism, tryptophan metabolism, and histidine metabolism (Fig. 3B).

### The selection of biological markers

A multiple-variable ROC analysis was conducted to identify the optimal set of biological markers. Initially, the overall sample was randomly divided into a training set and a validation set. The training set was used to train the model, while the validation set was employed to assess the model’s predictive performance (Tables 1 and 2).

**TABLE 1:** Grouping data of each component in the training set^*a*^

	LTBI (*n* = 50)	HC (*n* = 50)	*P-*value
Gender (male)	32 (64.0%)	29 (58.0%)	0.539
Age (median, interquartile range)	17.0 (17.0–50.0)	17.0 (4.0–38.0)	0.167
QFT result	Positive	Negative	

^*a*^ HC referred to the healthy control group, and LTBI referred to the latent tuberculosis infection group. Age was represented as the mean (median–interquartile range). The *P*-value for gender was calculated using the chi-square test, and the *P*-value for age was calculated using the *t*-test.

**TABLE 2:** Grouping data of each component in the validation set^*a*^

	LTBI (*n* = 31)	HC (*n* = 35)	*P-*value
Gender (male)	15 (48.4%)	18 (51.4%)	0.805
Age (median, interquartile range)	17.0 (17.0–49.0)	21.0 (17.0–55.0)	0.382
QFT result	Positive	Negative	

^*a*^ HC refers to the healthy control group, and LTBI refers to the latent tuberculosis infection group. Age is represented as the mean (median–interquartile range). The *P*-value for gender is calculated using the chi-square test, and the *P*-value for age is calculated using the *t*-test.

**Fig 4:**
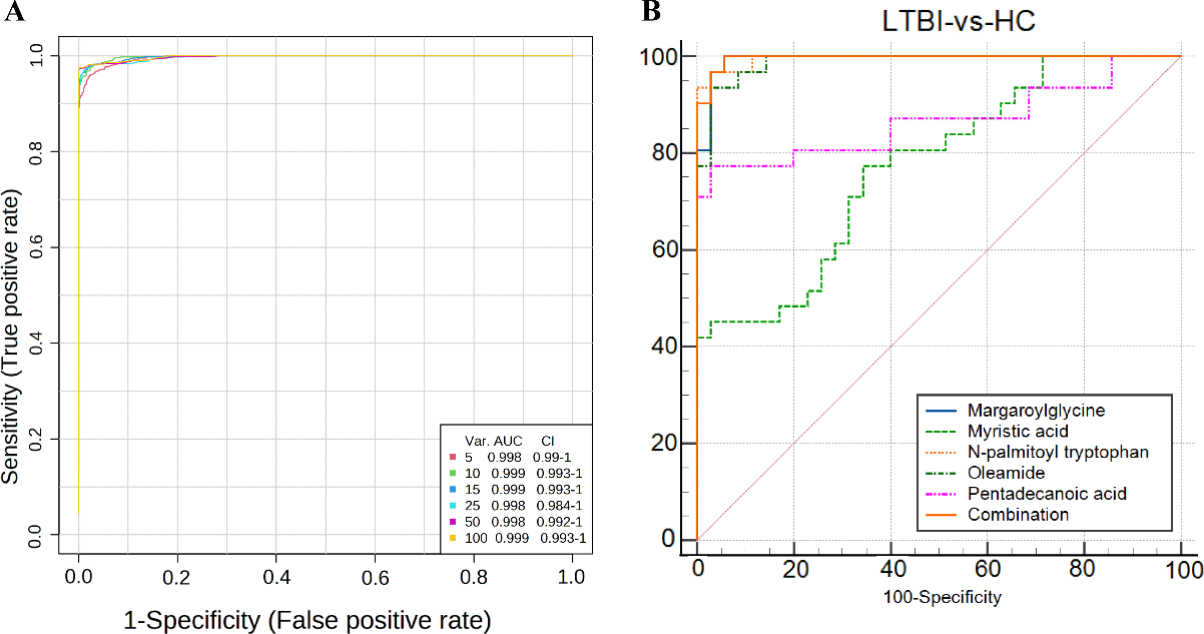
The selection of biological markers. (**A**) Multivariable ROC analysis. Based on the RandomForest classification method, variable importance is ranked according to the VIP values from PLS-DA. ROC curves and AUC values are provided for models using the top 5, top 10, top 15, top 25, top 50, and top 100 variables. (**B**) ROC analysis of 5 differential metabolites for LTBI Diagnosis in the validation set.

Metabolic profiling of the LTBI and HC groups was performed, integrating pathway analysis and biological function prediction. The RandomForest, SVM, and PLS-DA algorithms were then selected to identify the optimal marker set. A multiple classification approach was utilized to select metabolites and construct classification models through repeated sampling and cross-validation. This iterative process aimed to identify the optimal biological marker set. In the training set, the optimal variable combination was determined using the RandomForest algorithm, followed by cross-validation through repeated random sampling. During each cross-validation iteration, two-thirds of the samples were evaluated based on VIP scores, accuracy, RandomForest, or linear regression coefficients. The top two, three, five, ten, and up to one hundred variables were selected to build the classification or regression models, while the remaining samples were used for validation. The multiple ROC analysis revealed that the optimal biological marker set consisted of the top five metabolites identified through the RandomForest analysis, which were then ranked by their variable importance scores. The relative importance of each feature was assessed using the Gini importance measure. From this, the top two, three, five, ten, and one hundred most important metabolites were selected to construct classification and regression models. Their predictive performance was validated using the remaining one-third of the samples. ROC analysis plots revealed that the optimal biomarker panel, derived from the RandomForest analysis, was composed of the top five metabolites with the highest variable importance values (Fig. 4A). The five metabolites identified as potential biomarkers for distinguishing between LTBI and HC were as follows: margaroylglycine, N-palmitoyl tryptophan, oleamide, myristic acid, and pentadecanoic acid. A diagnostic model was then constructed using binary logistic regression to assess the accuracy of this biomarker panel in the validation set. The AUC was used to evaluate the model’s ability to discriminate between the LTBI and HC groups. In our discovery cohort, the combination of these five metabolites yielded a high AUC (0.996) (Table 3). However, we emphasize that this value likely reflects the optimized performance within a controlled study population. More importantly, the biological consistency of these markers—specifically the depletion of fatty acids and accumulation of amides/acylglycines—was successfully replicated in independent external cohorts (Fig. S6). This cross-cohort reproducibility suggests that the panel captures conserved metabolic responses to M.tb infection, supporting its potential utility beyond the specific performance metrics of the discovery set. The significant difference between the two groups was confirmed through statistical analysis. Finally, a classification model was constructed using the training set, and its diagnostic value was validated with binary logistic regression analysis. This validated the potential of these five metabolites as biological markers for distinguishing LTBI from HC (Fig. 4B). To ensure the reliability of the metabolite identification, we performed targeted MRM validation for three key lipid/fatty acid biomarkers. Using authentic standards, we successfully confirmed the identities of myristic acid, pentadecanoic acid, and oleamide by matching their retention times (8.44 min, 8.88 min, and 7.91 min, respectively) and specific MS/MS fragmentation patterns with the standards. The MRM chromatograms and mass spectra are provided in Fig. S3 to S5. Due to the limited commercial availability of standards for margaroylglycine and N-palmitoyl tryptophan at the time of the study, their annotations remain putative (Level 2 identification) based on high-confidence spectral matching with HMDB and MoNA databases.

**TABLE 3:** Evaluation of the diagnostic efficacy of LTBI using a logistic binary regression model

Variable	AUC	SE^*a*^	95% CI^*b*^
Margaroylglycine	0.994	0.00614	0.933 to 1.000
Myristic acid	0.772	0.0572	0.653 to 0.867
N-palmitoyl tryptophan	0.995	0.00444	0.937 to 1.000
Oleamide	0.988	0.00876	0.924 to 1.000
Pentadecanoic acid	0.866	0.0507	0.760 to 0.938
Combination	0.996	0.00363	0.938 to 1.000

^*a*^ The method of DeLong et al. (14) is used to calculate the standard error of the area under the curve (AUC).

^*b*^ The confidence interval for the AUC is calculated as AUC ± 1.96 SE (standard error).

We validated the identified metabolic signatures in independent external cohorts to confirm their reproducibility across different platforms and populations (Fig. S6). Consistent with our discovery phase, myristic and pentadecanoic acid exhibited sustained downregulation in the external GC-MS cohort (mean AUC = 0.69). In the independent LC-MS cohort (ST002428), we confirmed the diagnostic value of specific lipid accumulation: hexadecanamide (validating fatty acid amides) achieved an AUC of 0.74, while hexanoylglycine (serving as a proxy for acylglycines such as margaroylglycine) showed significant diagnostic accuracy (AUC = 0.76), reinforcing the hypothesis of incomplete fatty acid oxidation. Furthermore, the tryptophan pathway—precursor to N-palmitoyl tryptophan—demonstrated high diagnostic potential (AUC = 0.89), collectively verifying the biological robustness of these core metabolic features.

### Transcriptomic and metabolic profiling in tuberculosis latent infection

**Fig 5:**
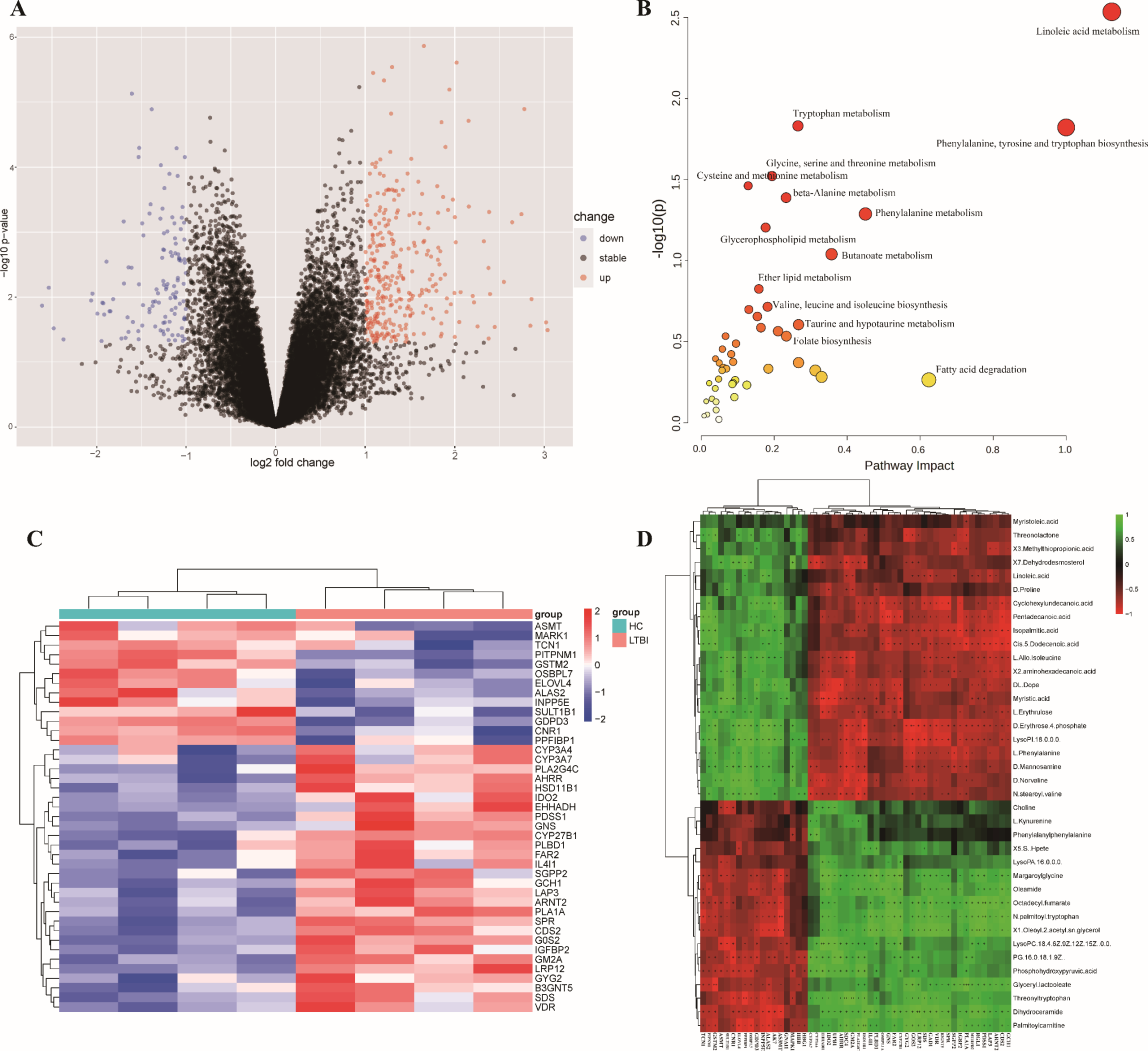
Transcriptomic and metabolic profiling in tuberculosis latent infection (**A**) volcano plot of differentially expressed genes. The horizontal axis represents the log2 fold change, which is the base-2 logarithm of the fold change in differential gene expression. The vertical axis represents the negative logarithm of the *P*-value from the *t*-test. Red points indicate significantly upregulated differentially expressed genes, blue points indicate significantly downregulated differentially expressed genes, and black points indicate genes with no significant difference. (**B**) Heatmap of differentially expressed genes. (**C**) Bubble plot of co-metabolic pathways analysis from transcriptomics and metabolomics. This plot shows the metabolic pathways enriched by differentially expressed metabolites and genes between the LTBI group and the HC group. Each bubble represents a metabolic pathway. The horizontal axis and bubble size indicate the impact factor of the pathway in the topological analysis, while the vertical axis and bubble color represent the *P*-value of the enrichment analysis (negative logarithm base 10). (**D**). Heatmap of the correlation analysis results between differentially expressed metabolites and genes between the LTBI group and the HC group. The horizontal axis represents differentially expressed genes, and the vertical axis represents differentially expressed metabolites. Green squares indicate positive correlations, while red squares indicate negative correlations. Lighter colors represent stronger correlations. Symbols represent significance levels: “+” indicates *P*-value < 0.1, “++” indicates *P*-value < 0.01, and “+++” indicates *P*-value < 0.001.

A search of the GEO database identified three datasets with relevant data on LTBI: GSE84445 (United States), GSE153342 (United Kingdom), and GSE98461 (China). Transcriptomic analysis was performed on all three datasets, and a consensus of differentially expressed genes was derived from each. However, two genes showed a low correlation with subsequent findings and lacked reports of involvement in infectious diseases. Given the genetic diversity among populations, GSE98461 was selected for further analysis. Differential genes identified from this data set were examined using the Joint Pathway Analysis module of the MetaboAnalyst 5.0 platform, which also facilitated the initial transcriptomic analysis. Bioinformatics analysis of GSE98461 identified 429 differentially expressed genes, with 311 upregulated and 118 downregulated (Fig. 5A). The expression trends of these genes were visualized in a heatmap (Fig. 5B). A comprehensive analysis of differential metabolites revealed significant enrichment in 47 metabolic pathways, including β-oxidation, tyrosine metabolism, tryptophan metabolism, and glycerophospholipid metabolism (Fig. 5C). To further explore the relationship between the differential genes and metabolites, Spearman’s rank correlation coefficient and *P*-values for the shared metabolic pathways were calculated. The resulting heatmap (Fig. 5D) and network map (Fig. S1) indicated the correlation between differential genes and metabolites. Subsequently, metabolite-mRNA pairs were filtered using a *P*-value threshold of 0.05 and a correlation coefficient greater than 0.8, identifying the strongest gene-metabolite associations. Several notable correlations were observed. A significant positive correlation was found between margaroylglycine and the genes *CYP27B1*, *EHHADH*, *IDO2*, *PLBD1*, and *IL4I1*, while N-palmitoyl tryptophan exhibited a significant negative correlation with *TCN1*. Oleamide showed a positive correlation with *IDO2*, whereas myristic acid negatively correlated with *CYP27B1*. Additionally, an unspecified acid indicated a significant positive correlation with *SULT1B1*, *CNR1*, and *ASMT* and a negative correlation with *CYP27B1*, *IDO2*, and *EHHADH*. Pentadecanoic acid was positively correlated with *TCN1* and *GSTM2* but negatively correlated with *FAR2*, *PDSS1*, *ARNT2*, *GCH1*, *IGFBP2*, *CYP27B1*, *CDS2*, and *GNS*.

### Analysis of the association between differential genes and related metabolites in the metabolic pathways of interest and validation of target genes

**Fig 6:**
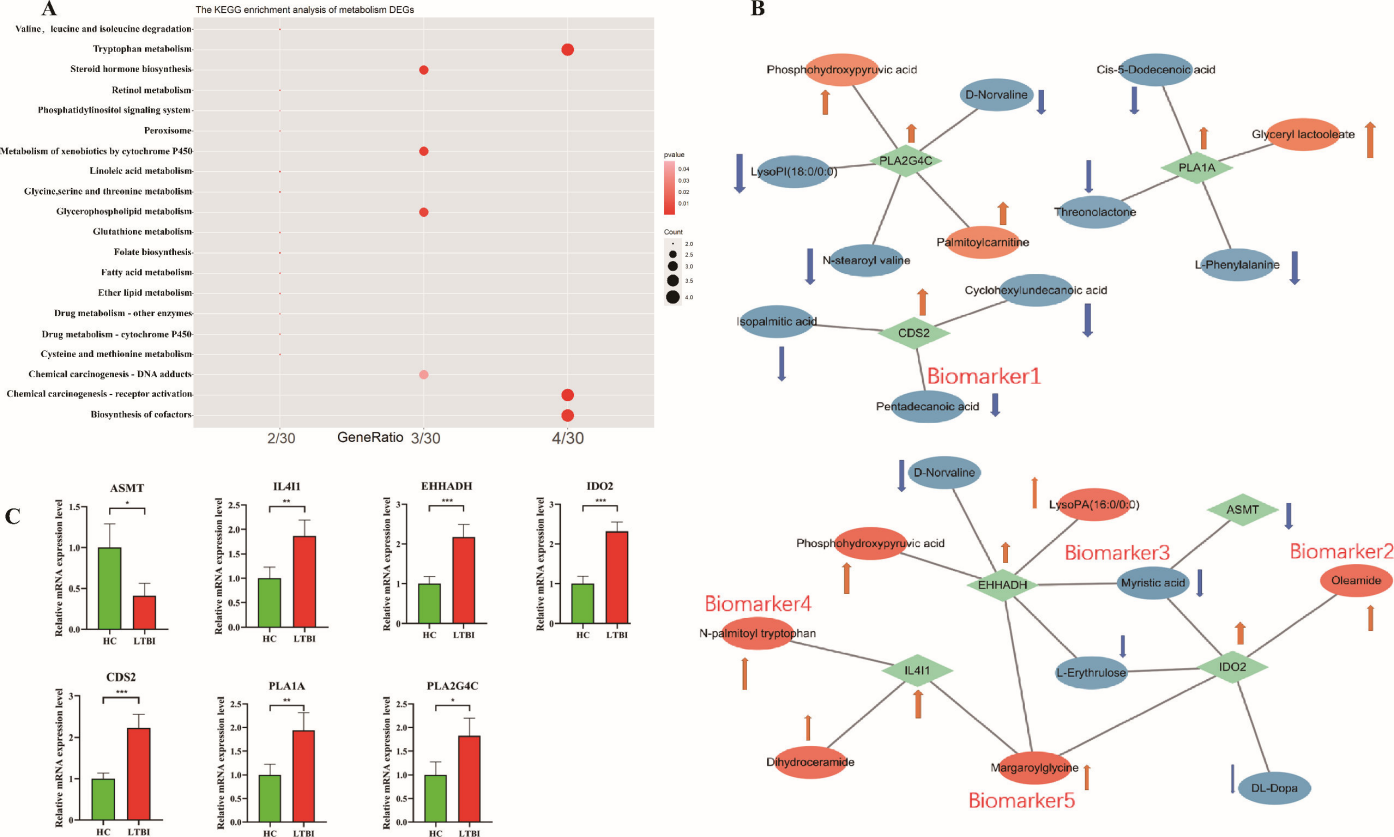
Analysis of the association between differential genes and related metabolites in the metabolic pathways of interest and validation of target genes. (**A**) KEGG pathways of differentially expressed genes in the co-metabolic pathway. The horizontal axis represents GeneRatio, which is the ratio of the number of differentially expressed genes associated with a given ID to the total number of genes in that term. The vertical axis represents metabolic pathways. The shade of red represents the *P*-value, with a darker color indicating a smaller *P*-value. (**B**) Gene-metabolite interaction networks in the glycerophospholipid metabolism pathway and tryptophan metabolism pathway. These networks are based on interactions between differentially expressed genes and differentially expressed metabolites. In the network, green diamonds represent differentially expressed genes in the pathway, located at the center of the network. Circles represent significantly related differentially expressed metabolites, distributed around the genes. Orange circles indicate significantly positively correlated metabolites, while blue circles indicate significantly negatively correlated metabolites. An orange arrow indicates that the gene/metabolite is upregulated in the organism, and a blue arrow indicates that the gene/metabolite is downregulated in the organism. (**C**) Real-time quantitative PCR detection of glycerophospholipid metabolism and tryptophan metabolism-related genes in PBMCs. The results are expressed as mean ± SEM, with a sample size of *n* = 8 for each group. Compared with the HC group, “*” indicates *P* < 0.05,“**” indicates *P* < 0.01, and “***” indicates *P* < 0.001.

To further investigate the metabolite-mRNA pairs identified in the combined metabolic pathways, a KEGG pathway enrichment analysis was conducted (Fig. 6A). The analysis revealed that the differential genes were predominantly enriched in pathways related to amino acid metabolism, steroid hormone biosynthesis, oleic acid metabolism, and glycerolipid metabolism, which were selected for further exploration. These pathways, in conjunction with nontargeted metabolic pathways, were analyzed to identify the most significant alterations in the transcriptomic and metabolomic profiles between LTBI and HC groups. The co-expression network diagram (Fig. 6B) for target genes and associated metabolites within these pathways highlighted key correlations. *PLA1A*, involved in glycerophospholipid metabolism, showed a significant positive correlation with glyceryl lactooleate and a significant negative correlation with threonolactone, L-phenylalanine, and cis-5-dodecenoic acid. *PLA2G4C* demonstrated a significant positive correlation with palmitoylcarnitine and phosphohydroxypyruvic acid, while negatively correlating with D-norvaline, lysoPI (18:0/0:0), and N-stearoyl valine. *CSD2* was significantly negatively correlated with cyclohexylundecanoic acid, pentadecanoic acid, and isopalmitic acid. In tryptophan metabolism, *IDO2* exhibited a significant positive correlation with margaroylglycine, lysoPC (18:4(6Z,9Z,12Z,15Z)/0:0), and oleamide and a negative correlation with L-erythrulose, DL-dopa, and myristic acid. *EHHADH* was positively correlated with lysoPC (18:4(6Z,9Z,12Z,15Z)/0:0), margaroylglycine, phosphohydroxypyruvic acid, and lysoPA (16:0/0:0) and negatively correlating with D-norvaline, L-erythrulose, and myristic acid. *IL4I1* showed positive correlations with N-palmitoyl tryptophan, dihydroceramide, and margaroylglycine. *ASMT* exhibited a significant positive correlation with myristic acid. These results highlight significant alterations in glycerolipid and oleic acid metabolism, as well as tryptophan metabolism, in both transcriptomic and metabolomic profiles between the two groups. Detailed information on these metabolic pathways is provided in Supplemental figure.

Building on the association analysis between differential genes and metabolites in glycerophospholipid and tryptophan metabolism, real-time quantitative PCR was employed to assess changes in the transcription levels of seven genes—*ASMT*, *IL4I1*, *EHHADH*, *IDO2*, *PLA2G4C*, *PLA1A*, and *CDS2*—in PBMCs from LTBI and HC groups. Compared to the HC group, the expression levels of genes associated with glycerophospholipid metabolism, particularly *PLA2G4C*, *PLA1A*, and *CDS2*, were significantly elevated, while *ASMT*, linked to tryptophan metabolism, was significantly reduced. Additionally, the expression levels of *IL4I1*, *EHHADH*, and *IDO2* were notably increased (Fig. 6C). These results corroborate that the observed alterations in glycerophospholipid and tryptophan metabolism in patients with LTBI are likely mediated or influenced by these genes.

## DISCUSSION

TB, the second deadliest infectious disease following COVID-19, ranks as the 13th leading cause of death worldwide. LTBI constitutes a major reservoir for new TB cases, with an estimated 1.7 billion people globally infected with M.tb. A primary contributor to the high incidence of pulmonary TB is the failure to detect and manage a significant proportion of individuals with LTBI in a timely manner. This study selected 85 healthy individuals and 81 patients with LTBI, identified through QFT results from close contacts of active patients with TB. Metabolomics was employed to identify 5 representative metabolites—margaroylglycine, N-palmitoyl tryptophan, oleamide, myristic acid, and pentadecanoic acid—and their diagnostic performance in distinguishing LTBI from healthy controls was assessed using ROC curves. The results revealed that these differential metabolites are involved in the pathological processes of pulmonary TB and reflect the underlying pathogenic mechanisms of M.tb.

Margaroylglycine, an acylglycine featuring a C-17 fatty acid group, is a minor metabolite of fatty acids. Elevated acylglycine levels are typically linked to fatty acid oxidation disorders and serve as biomarkers for these conditions, including in newborn screening (15, 16). In this study, patients with active pulmonary TB exhibited heightened energy conversion processes, such as the tricarboxylic acid cycle and oxidative phosphorylation. Upon entering macrophages, M. tb shifts its primary carbon and energy source from carbohydrates to lipids (17). The upregulation of margaroylglycine may reflect the bacterium’s utilization of host-derived carbon sources to sustain its replication and virulence, consistent with the hypothesis of accelerated lipid degradation and disturbances in the host’s energy metabolism.

N-palmitoyl tryptophan, an N-acylamide compound, is the palmitic acid amide of tryptophan and can be synthesized endogenously by the gut microbiota. In pediatric inflammatory bowel disease, N-palmitoyl tryptophan serves as both a clinical and inflammatory marker (18). This suggests that M.tb infection may induce an inflammatory response through an interaction with TRP channels and activation of GPCRs *via* N-palmitoyl tryptophan.

Oleamide, an endogenous fatty acid amide structurally related to the endocannabinoid anandamide, was initially noted for its sleep-inducing effects but is now recognized for its broad neuroprotective properties. Oleamide activates the NLRP3 inflammasome in primary monocyte-derived macrophages (MDMs), promoting M1 macrophage polarization and enhancing IL-1β production (19). It also inhibits LPS-induced inflammation by suppressing the mitogen-activated protein kinase (MAPK) and NF-κB signaling pathways in macrophages, paralleling the inflammatory responses observed in patients with ATB. We hypothesize that M.tb infection might provoke immune-inflammatory responses through mechanisms similar to oleamide-mediated modulation of inflammatory factors, although direct pathway validation requires further study.

Myristic acid, a 14-carbon saturated fatty acid found in various plant and animal fats, including common human foods, has been shown to mitigate testicular inflammation and apoptosis induced by hyperglycemia in diabetes, offering protective effects on the testes (20). In diabetic patients, myristic acid increases diacylglycerol kinase (DGK) δ protein levels and enhances glucose uptake in myotubes in a DGKδ-dependent manner (21). Additionally, it exhibits anti-inflammatory effects *in vitro*, promoting IL-10 production in LPS-stimulated macrophages (22). These findings imply that myristic acid could potentially serve as an anti-inflammatory regulatory factor in patients with LTBI and contribute to energy conversion processes, with its levels potentially decreasing during inflammatory states.

Pentadecanoic acid (C15:0), an essential odd-chain saturated fatty acid, offers notable benefits for cardiometabolic health, immunity, and liver function. It activates AMPK and inhibits mTOR, key regulators of the longevity pathway, and has been proposed as a potential biomarker for dairy fat intake and type 2 diabetes (T2D) risk (23, 24). In sarcopenia, pentadecanoic acid influences disease progression by activating AKT1 phosphorylation of NCOR1 and inducing FOXM1-mediated apoptosis (25). The reduced levels of pentadecanoic acid in patients with LTBI may be associated with disruptions in glucose metabolism caused by M.tb infection. Pulmonary TB often exacerbates diabetes by impairing glucose metabolism, potentially through systemic inflammation and the impact of anti-TB drugs on glucose regulation. Therefore, alterations in myristic and pentadecanoic acid levels in patients with LTBI may be associated with lipid and glucose metabolism disturbances linked to TB infection.

The metabolic pathways identified in this study are predominantly enriched in amino acid and lipid metabolism. Aromatic amino acids, including L-tryptophan, L-phenylalanine, and L-tyrosine, are essential for M.tb growth. Infected cells show that M.tb synthesizes phenylalanine and tyrosine *de novo via* selective bacterial enzymes. The chorismate mutase enzyme (M.tbCM), encoded by the gene *Rv1885c*, catalyzes the formation of these essential amino acids in the shikimate pathway. Khanapur et al. identified this enzyme as essential for TB pathogenesis, with its inhibition disrupting nutrient supply to M.tb (26, 27). While chorismate mutase is present in bacteria, fungi, and plants, it is absent in humans, making it a promising target for anti-TB drug development due to its unique bacterial sequence.

Through integrated transcriptomic and metabolomic analysis, significant alterations in glycerophospholipid metabolism and tryptophan metabolism pathways were identified in patients with LTBI, with strong correlations observed between candidate biomarkers and differential genes within these pathways. López-Hernández et al. reported changes in glycerophospholipid metabolism in T2D individuals with TB, including increased levels of LysoPC (18:1) and LysoPC (18:0) and decreased phosphatidylcholine concentrations (28). Phosphocholine and lysophosphatidylcholine, which transport fatty acids, phosphates, glycerol, and choline, were reduced, potentially limiting choline availability, a key nutrient and precursor for acetylcholine. These lipid alterations may influence cell recognition and signal transduction, contributing to significant weight loss in patients with LTBI. Additionally, clinical metabolomics studies have shown that serum and plasma tryptophan levels decline in patients with LTBI who progress to ATB, reflecting enhanced activity of indoleamine 2,3-dioxygenase (IDO), an enzyme upregulated in the lungs of TB-infected animals and involved in tryptophan catabolism (29). IDO-mediated tryptophan degradation, marked by increased kynurenine levels, plays a role in regulating CD4+ T cell responses and maintaining immune balance against M.tb infection. In our study, kynurenine, a metabolite differentially upregulated in the LTBI group, suggests that the changes in tryptophan metabolism in patients with LTBI are primarily driven by increased tryptophan catabolism into kynurenine.

PLA1A, PLA2G4C, and CDS2 are key regulators of the glycerophospholipid metabolism pathway. PLA1A and lysoPS are involved in the maturation and function of various immune cells, including T cells, dendritic cells, macrophages, and mast cells (30). In patients with LTBI, PLA1A may contribute to metabolic disturbances by affecting immune cell maturation and lysoPS production. PLA2G4C exhibits a nonclassical inflammatory profile, increasing plasma levels of arachidonic acid-derived eicosanoids and pro-inflammatory cytokines, such as IL-6, IL-8, MCP-1, and TNF-α (31, 32). The elevated transcription levels of *PLA2G4C* in patients with LTBI suggest that this gene plays a role in the development of systemic inflammation. *CDS2*, a novel regulator of lipid storage, modulates phosphatidic acid levels on lipid droplet surfaces, with a stronger effect than *CDS1* (33–36). The upregulation of *CDS2* in patients with LTBI aligns with the concept that M.tb might accelerate the utilization of lipid droplets upon entry into the human body to increase its energy supply.

*ASMT*, *IL4I1*, *EHHADH*, and *IDO2* are involved in tryptophan metabolism and show notable expression changes in patients with LTBI. The downregulation of *ASMT* suggests that M.tb infection may induce hormonal dysregulation by disrupting host-related metabolic pathways, which warrants further investigation regarding its potential link to clinical symptoms such as insomnia observed in some patients with TB. *IL4I1*, which has immunomodulatory functions, inhibits T cell activation and may regulate M2 macrophage polarization (37). Its upregulation in patients with LTBI indicates a role in immune evasion. *EHHADH*, which catalyzes two of the four reactions involved in the oxidation of long-chain fatty acids (38), is upregulated in patients with LTBI, indicating metabolic dysfunction in energy conversion processes. *IDO2*, which catalyzes the rate-limiting step in tryptophan degradation, is also upregulated in patients with LTBI. Studies suggest that *IDO2* expression in B cells regulates autoimmune responses by supporting the interaction between autoreactive T cells and B cells, with its expression necessary for a strong inflammatory response (39, 40). The upregulation of *IDO2* suggests that the IDO-kynurenine pathway contributes to the inflammatory response in patients with LTBI by inducing *IDO2* expression and promoting tryptophan catabolism.

A nontargeted metabolomics study of 6 samples that developed ATB identified 68 metabolites, with 44 downregulated and 24 upregulated. By comparing differential metabolites between the ATB/LTBI and LTBI/HC groups, 3 common metabolites were identified: stearolic acid and pentadecanoic acid levels decreased in patients with LTBI and further declined as the disease progressed, while phenylalanylleucine levels increased in patients with LTBI and continued to rise with disease progression. Although the small sample size limited data reliability and large-scale validation was not yet feasible, these findings provide a basis for future research into tracking LTBI progression to ATB and suggest potential early indicators for LTBI. With larger-scale studies, the progression from LTBI to ATB could be better understood. Despite the high sensitivity of our metabolomics equipment, which detected more metabolites than previous studies, the total number of identified metabolites remained limited, and some valuable metabolite information may have been overlooked. Thus, while small-molecule metabolites show promise as potential clinical biomarkers, further methodological refinements are necessary to enhance detection capabilities.

We acknowledge several limitations in our study. First, a major challenge in metabolic biomarker discovery is the potential for overfitting due to population homogeneity and confounding by the “exposome,” including long-term dietary habits and gut microbiota composition. Although we implemented strict overnight fasting prior to sampling to minimize immediate postprandial variations, we could not control for habitual diet or microbiome diversity. Consequently, plasma metabolite levels—particularly fatty acids (e.g., myristic acid and pentadecanoic acid) and amides (e.g., N-palmitoyl tryptophan)—should be interpreted as reflecting the composite host metabolic state associated with M.tb infection, potentially involving complex host-pathogen-microbiome interactions, rather than establishing direct causality. However, the robust replication of our core metabolic signatures in geographically and nutritionally diverse external cohorts (ST002428 and Lau et al.) argues against these findings being mere artifacts of local dietary intake, suggesting a conserved host immune-metabolic response (10). Second, detailed clinical metadata, such as BMI and specific smoking history, were not comprehensively available for all participants due to the retrospective nature of the sample collection. We acknowledge that these lifestyle variables are known to influence metabolic profiles. However, it is important to note that our study population consisted of a geographically localized cohort with shared environmental and dietary backgrounds, which helps minimize interindividual variability driven by lifestyle factors to a significant extent. Furthermore, the strict exclusion of participants with comorbidities (e.g., diabetes) ensures that the identified metabolic signatures primarily reflect the host response to M.tb infection rather than confounding metabolic diseases. Third, regarding the molecular validation, the transcriptomic analysis was conducted on a relatively small subset of samples (*n* = 4 per group), and the subsequent qPCR validation included 8 biological replicates per group. We recognize that these limited sample sizes may restrict the statistical power to detect subtle changes. However, the large effect sizes observed in our qPCR analysis (Cohen’s d > 1.64) suggest that the primary metabolic and transcriptomic signatures identified in this study are prominent and robust. Therefore, while these results should be interpreted as exploratory—providing mechanistic clues such as the link between IDO activation and tryptophan metabolism—the substantial magnitude of the differential expression supports their validity. Finally, the classification of LTBI and healthy control groups relied on QFT-GIT results. While our grouping strictly adhered to current clinical guidelines, we acknowledge the inherent limitations of IGRAs, including potential false positives or negatives and the inability to distinguish recent infection from remote exposure. This potential misclassification could introduce noise into the metabolite-gene association analyses. Future prospective studies with larger, multicentric cohorts and integrated multi-omics approaches are warranted to further validate the clinical utility and biological mechanisms of the identified panel.

## Data Availability

The transcriptomic data set analyzed in this study is available in the NCBI Gene Expression Omnibus (GEO) under accession number GSE98461 (41). The validation metabolomic dataset is available in the Metabolomics Workbench under accession number ST002428 (42). The raw LC-MS/MS metabolomics data generated in this study have been deposited in the OMIX database (China National Center for Bioinformation/Beijing Institute of Genomics) under accession number OMIX015166 (associated BioProject: PRJCA058404).

## SUPPLEMENTAL MATERIAL

Supplemental figures spectrum.03482-25-s0001.docx Figures S1 to S6.

Table S1 spectrum.03482-25-s0002.xlsx The original data matrix of metabolic features identified in positive and negative ion modes.

Table S2 spectrum.03482-25-s0003.xlsx Detailed information of 115 differential metabolites distinguishing the LTBI group from the HC group.

Table S3 spectrum.03482-25-s0004.xlsx List of significant differential metabolites filtered by False Discovery Rate (FDR) correction.

ASM does not own the copyrights to Supplemental Material that may be linked to, or accessed through, an article. The authors have granted ASM a non-exclusive, world-wide license to publish the Supplemental Material files. Please contact the corresponding author directly for reuse.
