# Supplementary material for: Integrated metabolomic and transcriptomic analysis reveals novel plasma biomarkers and metabolic pathway dysregulation in latent tuberculosis infection
Source: Microbiol Spectr. 2026 Mar 27;14(5):e03482-25. doi: 10.1128/spectrum.03482-25 (PMC13141921; doi:10.1128/spectrum.03482-25)
Supplement: Supplemental figures — Figures S1 to S6. [file spectrum.03482-25-s0001.docx]

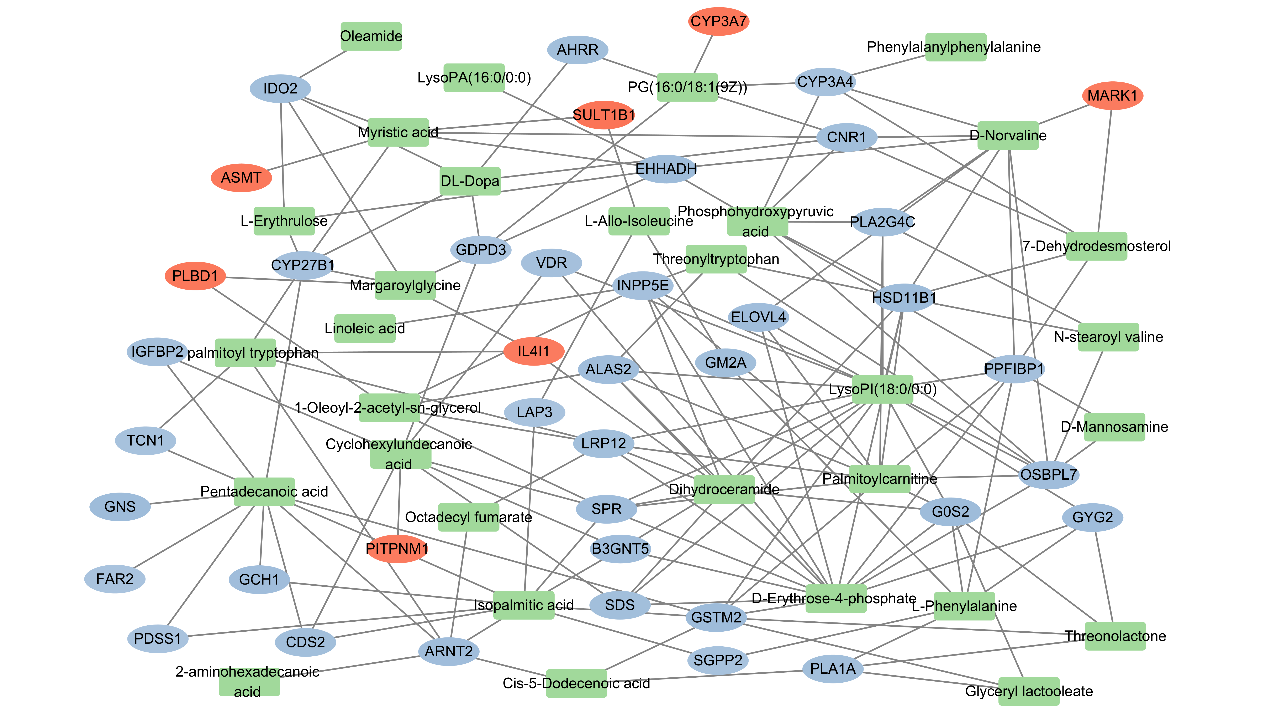


Supplementary Figure 1.**Correlation network diagram of transcriptomics and metabolomics analysis. This network illustrates the co-expression between differentially expressed genes and differentially expressed metabolites. In the network, green squares represent metabolites, located at the center of the network, while circles represent significantly related differentially expressed genes, distributed around the metabolites. Red circles indicate genes that are significantly positively correlated, and blue circles indicate genes that are significantly negatively correlated.**


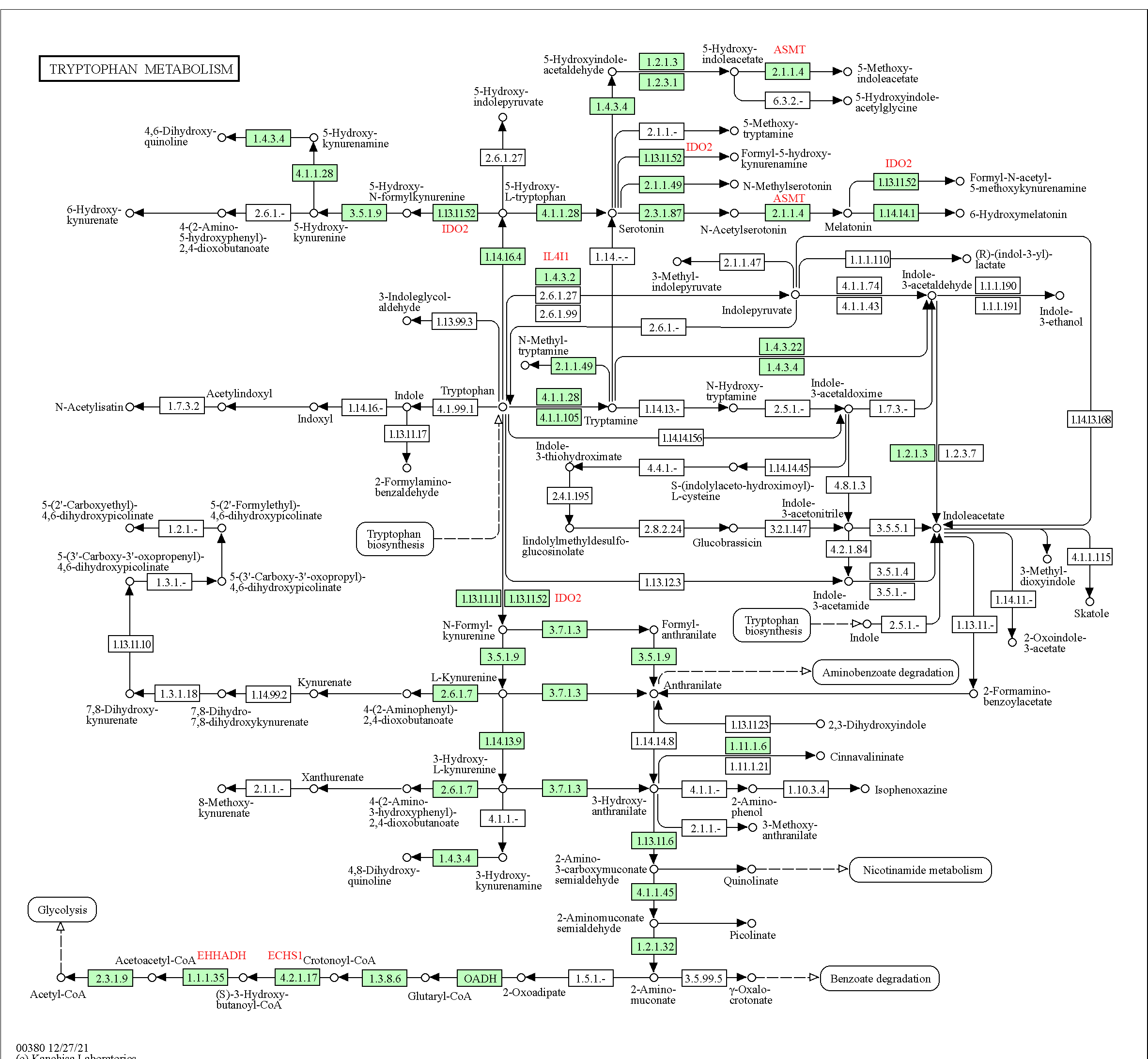

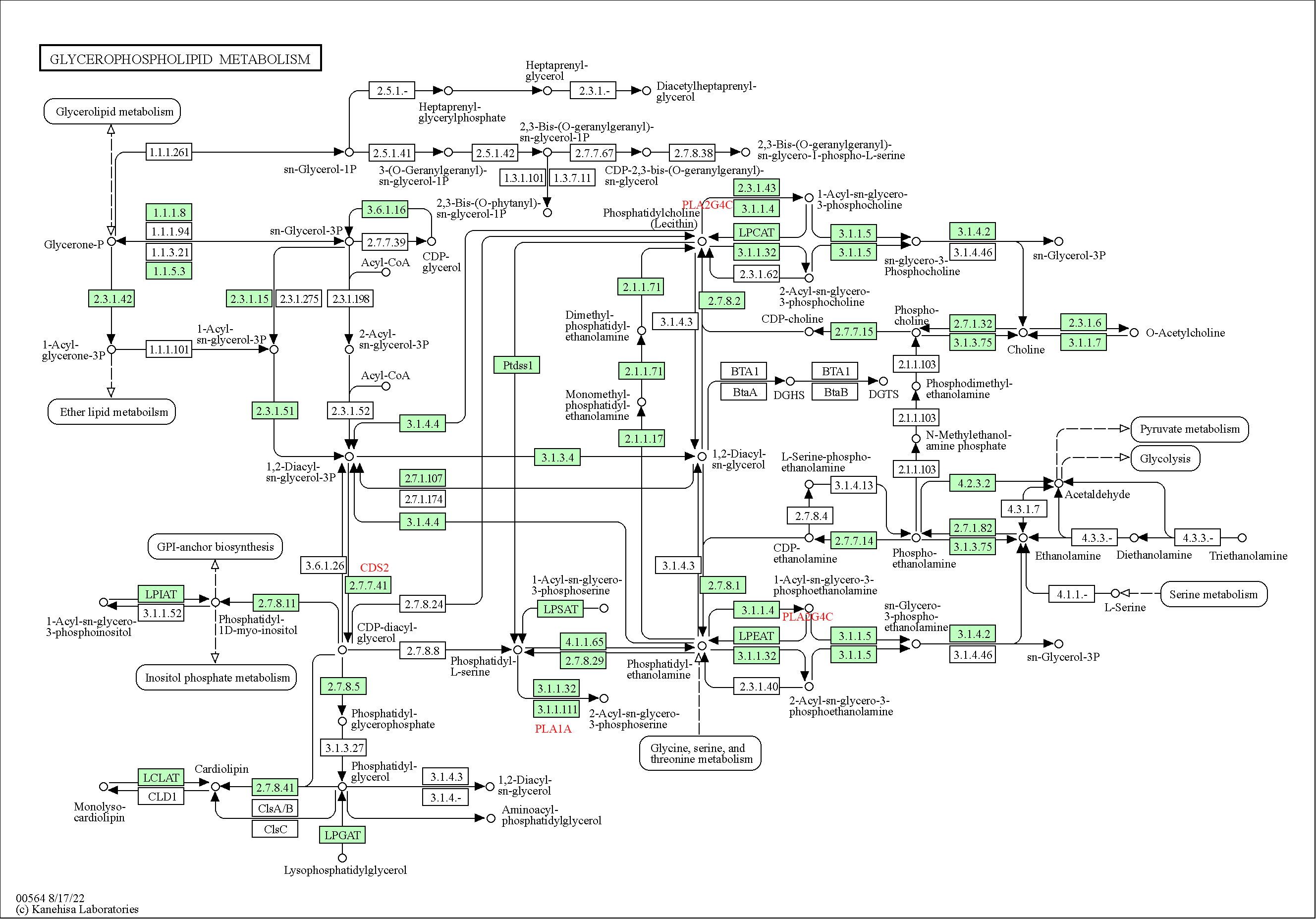


Supplementary Figure 2.**KEGG pathway map of differentially expressed genes in** **tryptophan metabolism and glycerophospholipid metabolism pathways.**


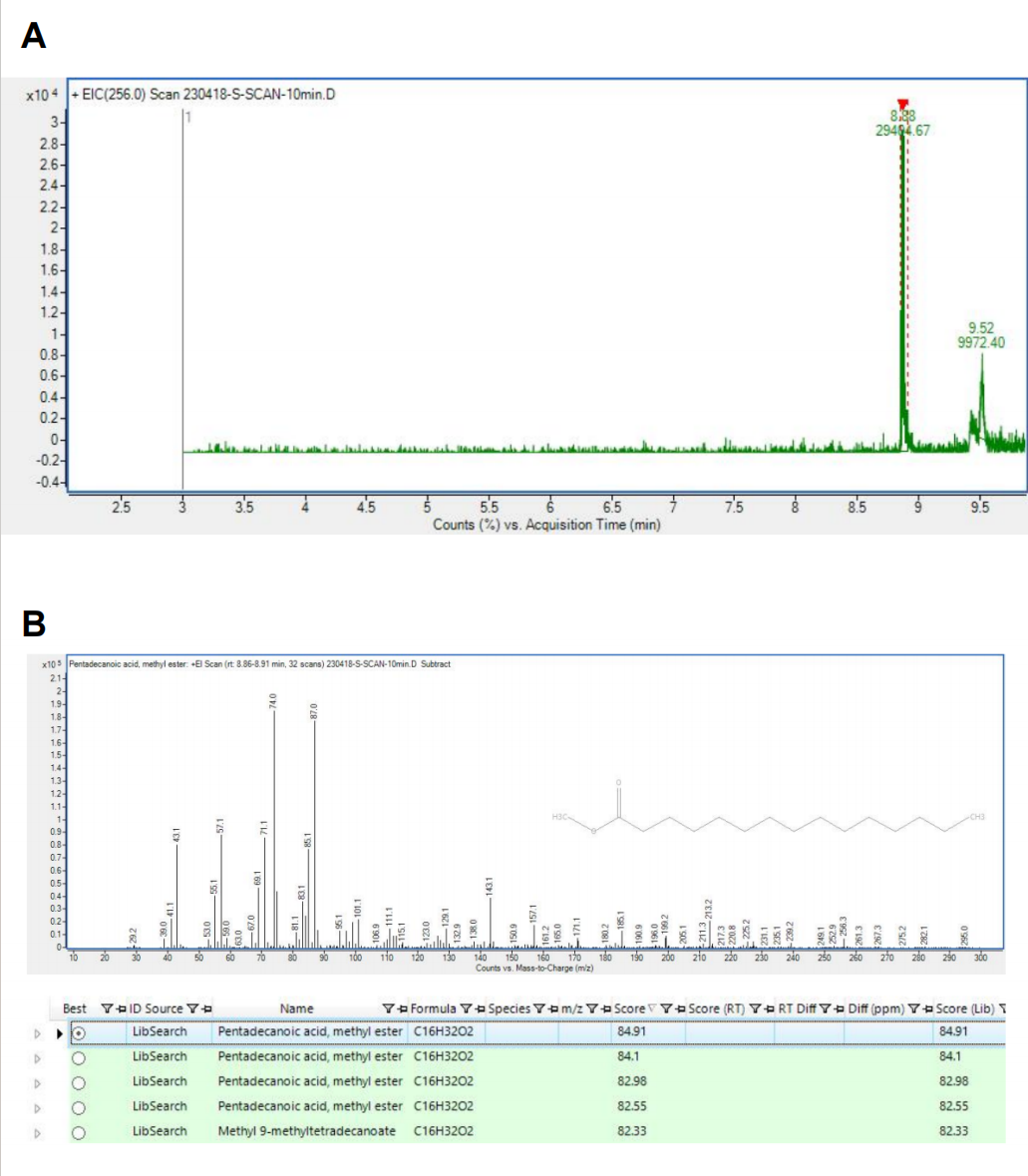


Supplementary Figure 3.**Targeted validation of Pentadecanoic acid. (A) Extracted Ion Chromatogram (EIC) of the authentic standard (m/z 256.0), showing a retention time of 8.88 min. (B) MS/MS spectrum matching with the reference library (NIST/HMDB), confirming the structural identity.**


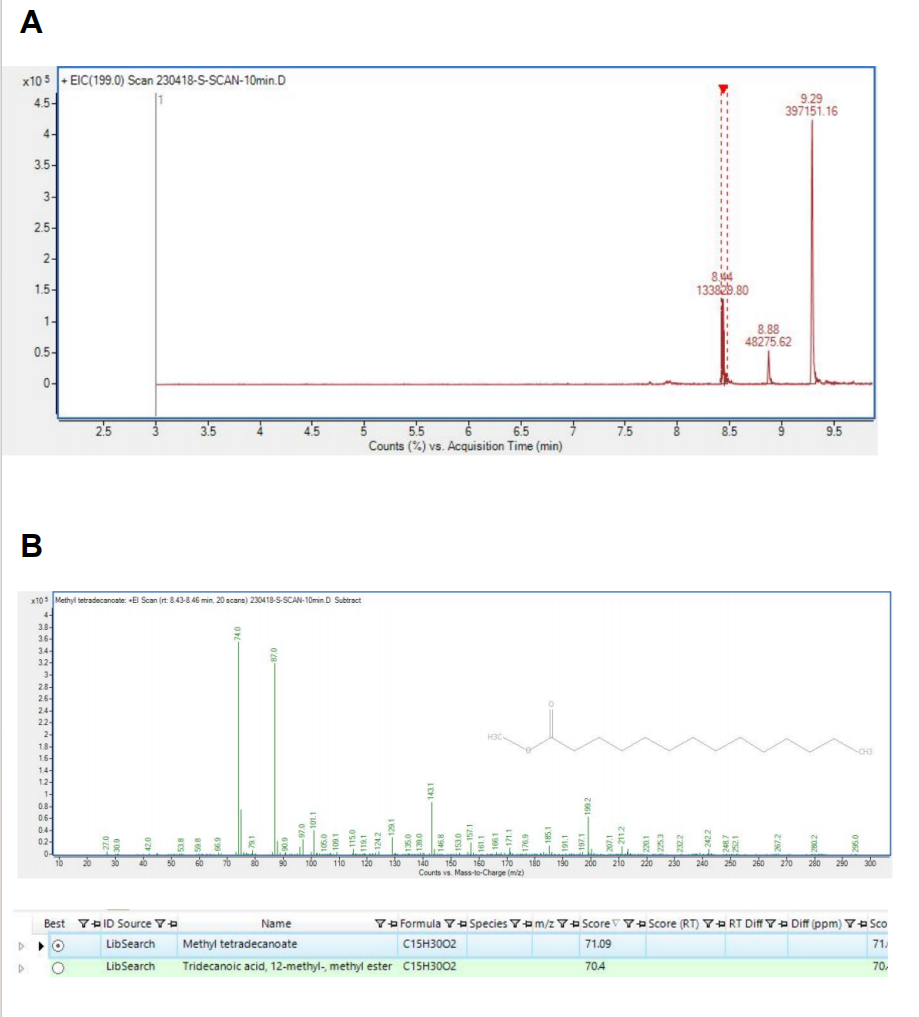


Supplementary Figure 4.**Targeted validation of Myristic acid. (A) Extracted Ion Chromatogram (EIC) of the authentic standard (m/z 199.0), showing a retention time of 8.44 min. (B) Optimization of MRM transitions showing the precursor ion and product ions.**


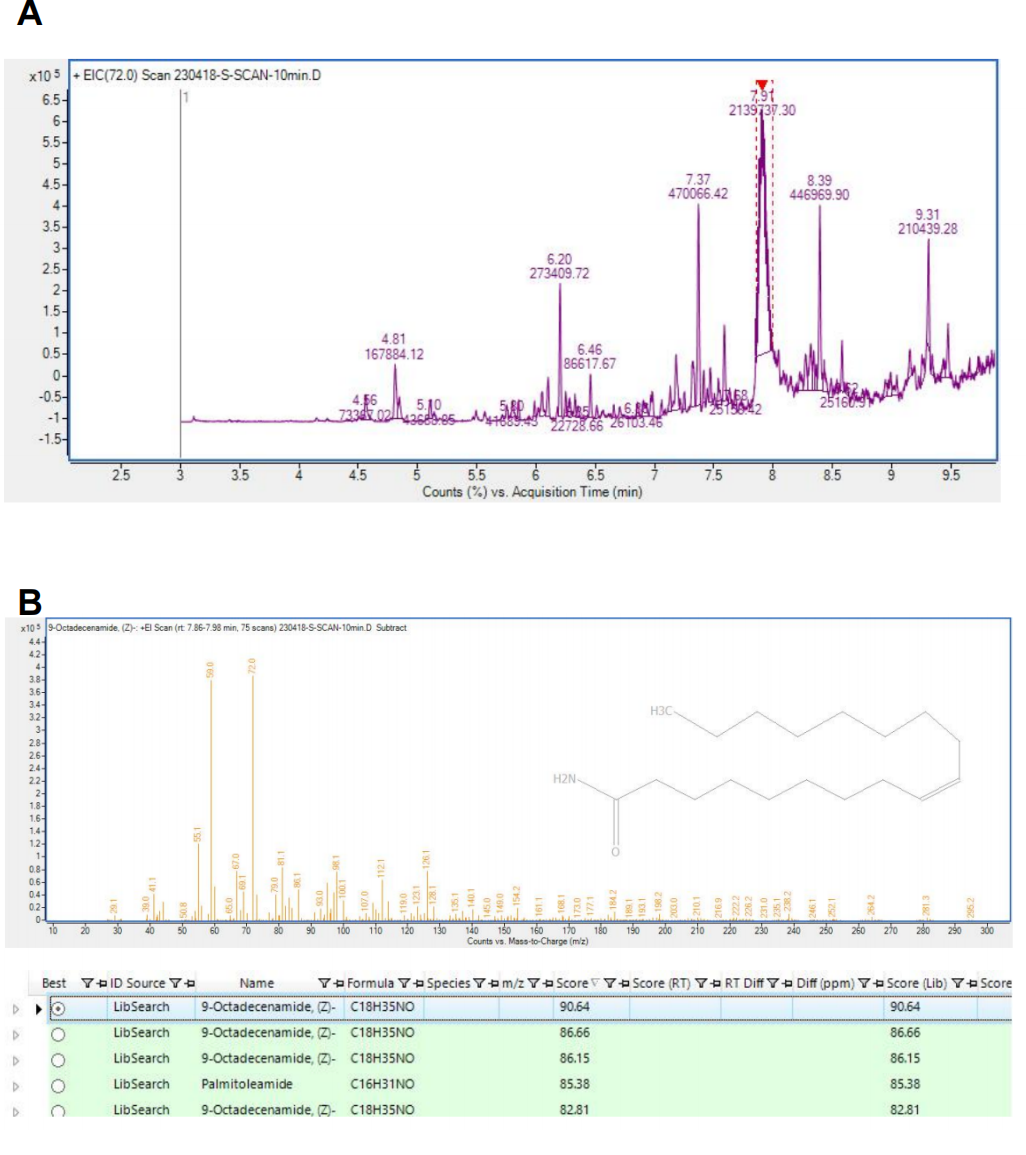


Supplementary Figure 5.**Targeted validation of Oleamide. (A) Extracted Ion Chromatogram (EIC) of the authentic standard (m/z 72.0), confirming a retention time of 7.91 min. (B) Spectral matching with the reference library, identifying the compound as Oleamide (9-Octadecenamide).**


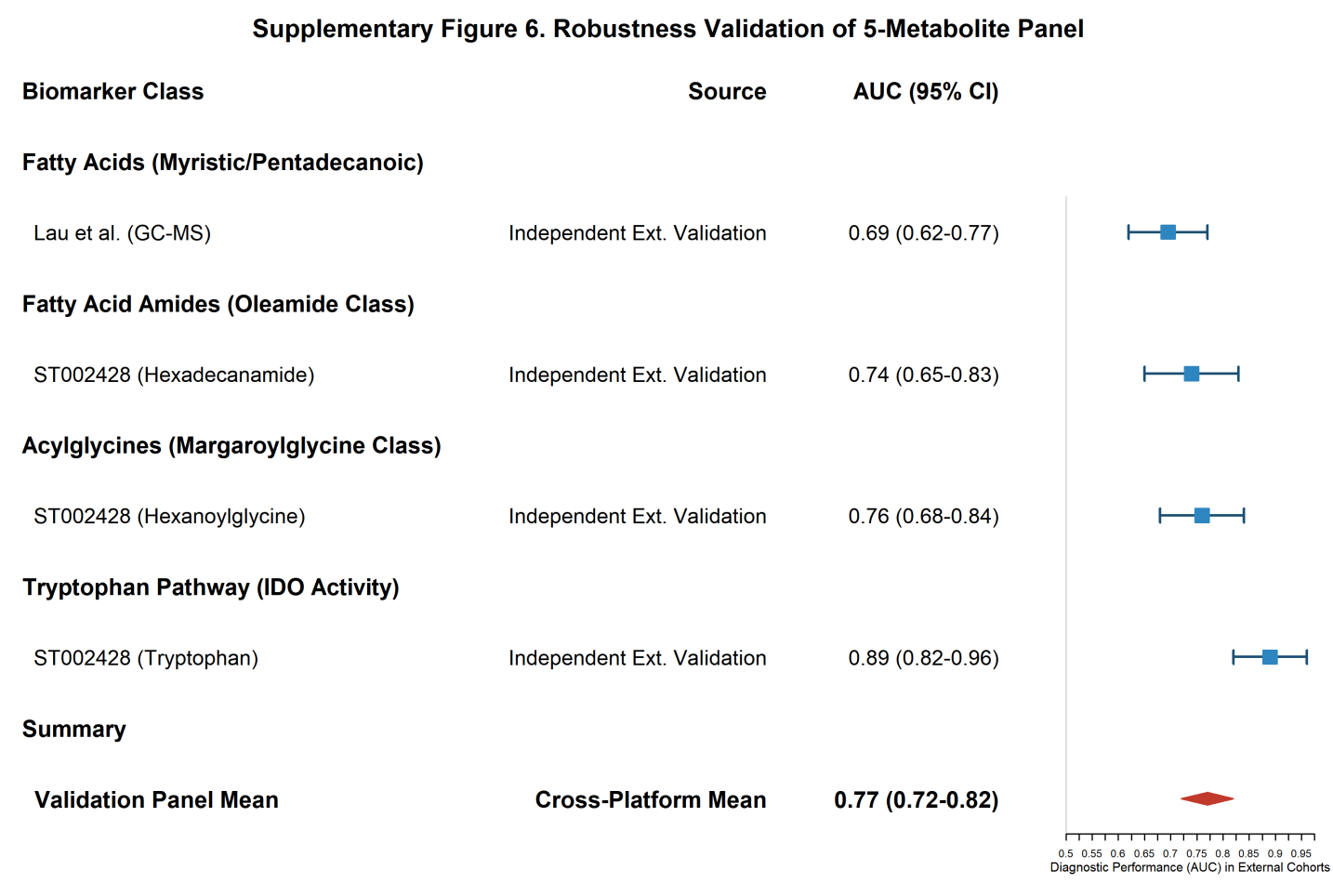


Supplementary Figure 6.**Forest plot summarizing external validation in independent cohorts. Diagnostic performance (AUC) was assessed for biomarkers or their class-specific proxies in independent datasets. Fatty acids (Myristic/Pentadecanoic) were validated in the Lau et al. cohort (Mean AUC = 0.69). In the ST002428 cohort, Hexadecanamide (Amide proxy, AUC = 0.74), Hexanoylglycine (Acylglycine proxy, AUC = 0.76), and Tryptophan (IDO pathway, AUC = 0.89) demonstrated robust diagnostic value. Error bars indicate 95% confidence intervals.**
